# Genome Sequence of *Dehalobacter* sp. Strain TeCB1, Able To Respire Chlorinated Benzenes

**DOI:** 10.1128/genomeA.01681-16

**Published:** 2017-02-23

**Authors:** Ricardo Alfán-Guzmán, Haluk Ertan, Mike Manefield, Matthew Lee

**Affiliations:** a School of Biotechnology and Biomolecular Sciences, University of New South Wales, Sydney, Australia; b Department of Molecular Biology and Genetics, Istanbul University, Vezneciler, Turkey

## Abstract

*Dehalobacter* sp. strain TeCB1 was isolated from groundwater contaminated with a mixture of organohalides and is able to respire 1,2,4,5-tetrachlorobenzene and 1,2,4-trichlorobenzene. Here, we report its 3.13-Mb draft genome sequence.

## GENOME ANNOUNCEMENT

Chlorinated benzenes are a group of isomeric aromatic compounds extensively used in the production of pesticides, insecticides, heat transfer agents, dyes, and as additives for rubber products (1, 2). This group of compounds are common groundwater pollutants and probable carcinogens (3). Release of these compounds to the environment often occurs due to inadequate handling or disposal; e.g., 1,2,4,5-tetrachlorobenzene (TeCB) and 1,2,4-trichlorobenzene (TCB) have been found in crops, fish, groundwater, and air in the United States, Europe, and China. Both substances are not only probable carcinogens, but it has been shown that acute exposure may lead to kidney or liver damage in humans (4–6).

Detoxification of chlorinated benzenes can occur when they are used as terminal electron acceptors in microbial respiration. *Dehalococcoides mccartyi* strain CBDB1 is able to reductively dechlorinate hexachlorobenzene (HCB) to 1,3,5-TCB, 1,3-, and 1,4-dichlorobenzene (DCB) (7). In 2014, Nelson et al. (8) isolated two *Dehalobacter* strains and a highly enriched culture able to respire a variety of chlorinated benzenes. In this study, we present the genome sequence of *Dehalobacter* sp. strain TeCB1, which is able to respire 1,2,4,5-TeCB and 1,2,4-TCB, yielding 1,3- and 1,4-DCB. Strain TeCB1 was isolated from organohalide-contaminated groundwater in Sydney, Australia.

Genomic DNA from strain TeCB1 was extracted according to Murray et al. (9) and then sequenced using an Illumina HiSeq 2500 sequencer at Novogene Bioinformatics Technology (Beijing, China). A total of 2,341,965 high-quality 100-bp paired-end reads were generated (coverage 100×). *De novo* assembly was performed using SPAdes assembler version 3.6.1 standard pipeline (10), generating 67 contigs. Annotation of the assembled genome was conducted via the NCBI Prokaryotic Genome Annotation Pipeline (version 3.3). The *Dehalobacter* sp. strain TeCB1 complete genome is 3.13 Mb long, with *N*_50_ value of 105,922 bp, and comprises a G+C content of 44%; a total of 3,081 genes were discovered, in which 2,962 are protein-coding genes, seven were rRNA genes (i.e., three 5S, one 16S, and three 23S), and 50 were tRNA genes. A genome quality assessment tool (CheckM) was used to assess the quality and purity of the draft genome (11). It reported a completeness of 99.94% based on the finding of 418/420 lineage-specific marker genes (marker lineage *Clostridia*), contamination of 0.17%, and strain heterogeneity tested by the amino acid identity between multicopy genes of zero.

Although other strains able to respire chlorinated benzenes belonging to the genus *Dehalobacter* have been isolated, to date, only the draft genome sequence of strain TeCB1 is publicly available (https://www.ncbi.nlm.nih.gov/nuccore/1055168950).

Strain TeCB1’s genome was compared to that of its closest relative, *Dehalobacter restrictus* PER-K23; among the many differences found, TeCB1 possesses a single 16S rRNA gene, while the PER-K23 genome contains four (12). Strain TeCB1 encodes 24 reductive dehalogenase (RdhA) homologs, one of them N-terminally truncated and five unique to this strain, together with a complete set of genes for *de novo* cobalamin and menaquinone biosynthesis and the Wood-Ljungdahl pathway. The genome also comprises the genes encoding various kinds of [NiFe]- and [FeFe]-hydrogenases, including Hup-type and bifurcating hydrogenases.

### Accession number(s).

This whole-genome shotgun project has been deposited at DDB/ENA/GenBank under the version number MCHF00000000.
